# Evaluación de la formación de biopelículas en aislamientos bacterianos y fúngicos por el método semicuantitativo de microtitulación con cristal violeta y el cualitativo de agar con rojo Congo

**DOI:** 10.7705/biomedica.6732

**Published:** 2023-08-31

**Authors:** Xiomara Moreno, Melanie Ventura, María Mercedes Panizo, María Fátima Garcés

**Affiliations:** 1 Departamento de Microbiología, Instituto Médico La Floresta, Caracas, Venezuela Instituto Médico La Floresta Instituto Médico La Floresta Caracas Venezuela; 2 Cátedra de Bacteriología, Escuela de Bioanálisis, Facultad de Medicina, Universidad Central de Venezuela, Caracas, Venezuela Universidad Central de Venezuela Universidad Central de Venezuela Caracas Venezuela; 3 Escuela de Bioanálisis, Facultad de Medicina, Universidad Central de Venezuela, Caracas, Venezuela Universidad Central de Venezuela Universidad Central de Venezuela Caracas Venezuela; 4 Servicios Hospitalarios MCG, Caracas, Venezuela Servicios Hospitalarios MCG Caracas Venezuela; 5 Laboratorio de Investigaciones Básicas y Aplicadas, Escuela de Bioanálisis, Facultad de Medicina, Universidad Central de Venezuela, Caracas, Venezuela Universidad Central de Venezuela Universidad Central de Venezuela Caracas Venezuela

**Keywords:** biopelículas, bacterias grampositivas, bacterias gramnegativas, levaduras, rojo Congo, Biofilms, Gram-positive bacteria, Gram-negative bacteria, yeasts, Congo red

## Abstract

**Introducción.:**

El 65 % de las infecciones humanas son producidas por bacterias o levaduras, cuya capacidad de formar biopelículas las hace más resistentes a los antimicrobianos y antifúngicos.

**Objetivo.:**

Determinar la capacidad de formación de biopelículas en aislamientos bacterianos y fúngicos por medio de los métodos cuantitativo de microtitulación con cristal violeta y cualitativo de cultivo en agar con rojo Congo.

**Materiales y métodos.:**

Con el método cuantitativo, se utilizaron los medios de cultivo infusión cerebro-corazón, tripticasa de soya y Müeller-Hinton para aislamientos bacterianos; para levaduras, se usaron caldo infusión cerebro-corazón y Sabouraud dextrosa. Para el método cualitativo de cultivo en agar, se utilizaron los mismos medios de cultivo más una solución con 3 % de rojo Congo y 10 % de dextrosa. Cómo método de referencia, se utilizó la propuesta de Stepanovic *et al*.

**Resultados.:**

Se evaluaron 103 aislamientos bacterianos y 108 de levaduras. No es recomendable sustituir el caldo infusión cerebro-corazón por los caldos tripticasa de soya y Müeller-Hinton en el método cuantitativo, para evaluar la formación de biopelículas en los aislamientos bacterianos. El medio Sabouraud dextrosa, en caldo y agar, puede sustituir al de infusión de cerebro-corazón para evaluar la formación de biopelículas en levaduras, tanto por el método cuantitativo como por el cualitativo.

**Conclusión.:**

El estudio de las biopelículas en el laboratorio de microbiología, a partir del método cualitativo de cultivo en agar con rojo Congo, es un procedimiento sencillo, rápido y de bajo costo, que proporciona información útil para el diagnóstico y la terapéutica de infecciones persistentes causadas por bacterias y levaduras.

## Abstract (en)

**Introduction.:**

Sixty-five percent of human infections are caused by bacteria or yeasts able to form biofilms. This feature makes them more resistant to antimicrobials and antifungals.

**Objective.:**

To determine biofilm formation capacity of bacterial and fungal isolates by quantitative crystal violet microtiter and qualitative Congo red agar methods.

**Materials and methods.:**

Brain-heart infusion, trypticase soy broth and Müeller-Hinton culture media were used in bacterial isolates for the quantitative method; brain-heart infusion broth and Sabouraud dextrose were used for yeasts. The same culture media plus 3% Congo red and 10% dextrose were used to apply the qualitative method in agar. The proposal by Stepanovic, *et al*. was used as a reference method.

**Results.:**

We evaluated 103 bacterial isolates and 108 yeasts isolates. We did not recommend substitute brain-heart infusion broth for trypticase soy and Müeller-Hinton broths for biofilm formation assessment in bacterial isolates using the quantitative method. Sabouraud dextrose medium, both broth and agar, can replace brain-heart infusion to assess biofilm formation in yeasts, quantitatively and qualitatively.

**Conclusion.:**

The study of biofilms in the microbiology laboratory, using Congo red agar qualitative method, is a simple, fast, and inexpensive procedure that provides precise information for the diagnosis and treatment of persistent infections caused by bacteria and yeasts.

Las biopelículas son comunidades de microorganismos infiltrados en una matriz de exopolisacáridos y adheridos a un substrato inerte o tejido vivo. Estas biopelículas están conformadas principalmente por bacterias y levaduras, son heterogéneas, aumentan la capacidad de sobrevivir de sus productores, presentan diversos microambientes en su interior, mantienen la comunicación entre los microorganismos e incrementan su resistencia a los antimicrobianos ^1^.

Los microorganismos productores de biopelículas han sido identificados como los agentes responsables de más del 65 % de las infecciones asociadas con la atención en salud y con las comunitarias, lo que representa un problema de salud pública mundial ^2^^-^^4^. La capacidad que tienen algunos microorganismos para formar biopelículas se ha asociado, también, con su capacidad para producir cuadros clínicos complejos. Diferentes infecciones, como la septicemia, la neumonía y la infección asociada con catéteres, prótesis y válvulas cardiacas, usualmente, son producto de la colonización por parte de microorganismos que contienen genes responsables de mecanismos de resistencia a los antimicrobianos ^5^^,^^6^.

Microorganismos como *Pseudomonas aeruginosa, Klebsiella pneumoniae* y *Staphylococcus aureus*, son responsables de neumonías hospitalarias e infecciones asociadas con dispositivos como prótesis y válvulas. Por otra parte, *K. pneumoniae*, además de las diversas especies de *Candida* y los estafilococos coagulasa negativos, suelen estar presentes en infecciones urinarias, vulvovaginales, dérmicas y de tejidos blandos. En todos los casos, estos microorganismos son capaces de generar cuadros clínicos graves de septicemia, difíciles de tratar con tratamientos antimicrobianos comunes ^1^^,^^4^^,^^7^^,^^8^. Por lo tanto, la detección de cepas productoras de biopelículas resulta de utilidad en el ámbito clínico, para orientar el tratamiento de elección de las enfermedades producidas por dichos microorganismos. Su detección temprana permite a los médicos tratantes establecer mejores estrategias terapéuticas, sabiendo que son patógenos persistentes y multirresistentes ^9^.

El método cuantitativo en microplaca con tinción de cristal violeta y el método cualitativo en agar Sabouraud dextrosa suplementado con rojo Congo, son dos de los más utilizados en el laboratorio de microbiología para la detección de biopelículas ^4^. El primero se considera de referencia, cuenta con gran sensibilidad, es semicuantitativo y permite clasificar las cepas como productoras fuertes, moderadas o débiles, o como no productoras de biopelículas. Sin embargo, es laborioso, consume mucho tiempo y requiere el uso de un espectrofotómetro ^10^^,^^11^. En cambio, el método de cultivo en agar con rojo Congo posee gran especificidad, es rápido, sencillo y de bajo costo. No obstante, presenta poca sensibilidad y, al ser cualitativo, no permite semicuantificar, ni clasificar la capacidad de formar biopelículas, como el método descrito anteriormente ^3^^,^^4^.

En diversos estudios se han comparado los resultados obtenidos con ambos métodos, con la finalidad de determinar si el agar con rojo Congo puede considerarse valioso para la detección temprana de cepas productoras de biopelículas ^8^^,^^12^^-^^17^.

En este sentido, el presente trabajo se propuso estudiar la capacidad de formación de biopelículas, mediante el método semicuantitativo de microtitulación con cristal violeta y el cualitativo por cultivo en agar con rojo Congo, de aislamientos bacterianos y fúngicos obtenidos a partir de muestras clínicas de diferentes sitios anatómicos, procedentes de pacientes que acudieron al Departamento de Microbiología del Instituto Médico La Floresta en Caracas (Venezuela) solicitando estudios microbiológicos.

## Materiales y métodos

### Aislamientos bacterianos y fúngicos

Se recolectaron aislamientos clínicos de bacterias y levaduras obtenidos mediante cultivo de muestras de diferentes sitios anatómicos de pacientes que solicitaron estudios microbiológicos en el Departamento de Microbiología del Instituto Médico La Floresta, desde el primero de marzo hasta el 11 de diciembre de 2020.

Los aislamientos se caracterizaron por la metodología manual convencional y por el método automatizado VITEK-2-Compact®, conservándose en agar nutritivo a temperatura ambiente hasta su procesamiento. Posteriormente, los aislamientos se subcultivaron en agar MacConkey (MCK-Oxoid®, GA, USA) para bacilos gramnegativos, agar colistina con ácido nalidíxico (CNA-Oxoid®, GA, USA) para cocos grampositivos, y agar Sabouraud dextrosa (SDG-Oxoid®, GA, USA) más gentamicina para levaduras. Estos subcultivos fueron incubados a 35 °C, de 24 a 48 horas, con el objetivo de verificar su viabilidad y pureza.

### Determinación de la capacidad de formación de biopelículas por el método semicuantitativo de microtitulación con cristal violeta

Los microorganismos en estudio se sometieron a diferentes medios de cultivo para comparar su rendimiento. Para las bacterias, se utilizó caldo infusión cerebro-corazón (BHI-Oxoid®, GA, USA) (medio de cultivo de referencia establecido por Stepanovic *et al*., 2000) ^10^, caldo Müeller- Hinton (Oxoid®, GA, USA) y caldo tripticasa soya (Oxoid®, GA, USA). Para las especies de Candida, se utilizó caldo infusión cerebro-corazón y caldo Sabouraud dextrosa (Oxoid®, GA, USA).

*Preparación del inóculo*. De los aislamientos obtenidos, previa verificación de viabilidad y pureza en agar MacConkey, colistina con ácido nalidíxico y Sabouraud dextrosa, se preparó una suspensión en 0,85 % de solución salina estéril, a una concentración de 0,5 McFarland (1-5 x 10^6^ UFC/ml).

Se tomaron 200 μl de dicha suspensión y se colocaron los aislamientos bacterianos en 19,8 ml de caldo de los medios infusión cerebro-corazón, Müeller-Hinton y tripticasa soya (grampositivos y gramnegativos); y los de levaduras, en Sabouraud dextrosa e infusión de cerebro-corazón, obteniendo así una dilución 1:100 de cada aislamiento ^3^^,^^4^.

*Microtitulación en placas de poliestireno*. Se utilizaron placas de poliestireno de 96 pocillos (Micro Test^TM^, Becton Dickinson Labware, NJ, USA).

En cada pocillo, se añadieron 200 ml de la dilución previa (1:100) de cada aislamiento: para las bacterias, provenientes de los caldos BHI, MH y TSB, y para las levaduras, provenientes de los caldos SDG y BHI.

Tanto para bacterias como para levaduras, se utilizó una placa para cada medio de cultivo y las pruebas se practicaron por triplicado. La primera fila de pocilios de cada placa se cargó solo con 200 μl de cada medio, sin ningún inóculo microbiológico, los cuales sirvieron como control negativo.

Todas las placas inoculadas se cubrieron con papel Parafilm® - BemisTM HS234526C (WI, USA) y se incubaron a 35 °C, las de bacterias por 24 horas y las de levaduras por 48 horas. Posteriormente, los caldos inoculados se decantaron y los pocillos se lavaron tres veces con solución tampón de fosfato a pH 7,4 (PBS), Fisher Bioreagents BP2438-4 (PA, USA). Se añadieron 200 μl de metanol (Merck, Darmstadt, Alemania) a cada pocillo para fijar la muestra, durante 30 minutos. El metanol se decantó y se repitió tres veces el lavado con PBS. A continuación, se agregaron 200 μl de cristal violeta al 1 % (Merck, Darmstadt, Alemania), por 30 minutos. Luego, el cristal violeta se decantó y el exceso se lavó con solución tampón fosfato salino. Posteriormente, se agregó a cada pocillo 200 μl de etanol al 96% (Merck, Darmstadt, Alemania) y se dejó reposar durante cinco minutos. La densidad óptica (DO) se leyó a 575 nm para las bacterias y a 490 nm para las levaduras, en un lector para ELISA (iMark^tm^, Microplate Reader, Bio-Rad, GA, USA) ^3^^,^^4^.

Para la interpretación de los resultados se utilizó la clasificación establecida por Stepanovic *et al*., adaptándola a microorganismos grampositivos, gramnegativos y levaduras, mediante la siguiente fórmula: X + (Σ x 3) ^11^; donde X= media de los blancos como control negativo; Σ= desviación estándar de las densidades ópticas obtenidas de los blancos; 3=desviaciones estándar. Según los cálculos, los aislamientos se clasificaron como: no formadores (NF), poco formadores (PF), moderadamente formadores (MF) y fuertemente formadores (FF) de biopelículas ^10^.

### Evaluación de los cambios de la pared celular implicados en la formación de la matriz de exopolisacáridos por el método cualitativo por cultivo en agar con rojo Congo.

Se preparó una suspensión de los aislamientos provenientes de la incubación por 24 horas en los agares MacConkey (grampositivos), colistina con ácido nalidíxico (gramnegativos) y Sabouraud dextrosa (levaduras), en 0,85 % de solución salina estéril. La suspensión se ajustó a un patrón de turbidez de 0,5 McFarland y, de esta, se tomaron 100 μl y se inocularon en placas con los agares con los medios infusión cerebro-corazón, Müeller-Hinton, tripticasa de soya y Sabouraud dextrosa para los microorganismos gramnegativos, grampositivos y levaduras, respectivamente. Esto se hizo para comparar su rendimiento. Todos los agares fueron suplementados con 3 % de rojo Congo y 10 % de dextrosa. Las suspensiones de cada asilamiento fueron inoculadas por triplicado usando la técnica de dilución por agotamiento y se incubaron a 35 °C entre 24 a 72 horas. La interpretación de la prueba se basó en la producción de un precipitado de color negro brillante de las colonias bacterianas o de un color rosado intenso a rojo en las levaduras del género Candida, que indica la producción de exopolisacáridos ^3^^,^^4^^,^^18^^,^^19^.

### Control de calidad

Se utilizaron como cepas de referencia de la *American Type of Culture Collection* (ATCC®): *Klebsiella pneumoniae* (70060), *Pseudomonas aeruginosa* (27853), *Staphylococcus aureus* (25923), *Candida albicans* (90028), *Candida tropicalis* (750), *Candida glabrata* (90030) y *Candida parapsilosis* (22019).

### Análisis estadísticos

Los datos obtenidos se organizaron en una tabla de Excel. Se calcularon la media aritmética y la desviación estándar de las lecturas de la densidad óptica obtenidas por el método semicuantitativo de microtitulación con cristal violeta, y se determinaron las frecuencias y los porcentajes para cada variable seleccionada.

Para evaluar la concordancia del método cualitativo por cultivo en agar con rojo Congo con el método semicuantitativo de microtitulación con cristal violeta (considerado como método de referencia) en los diferentes medios de cultivo, se utilizaron tablas de contingencia de 2 x 2 y el coeficiente kappa. La interpretación de los resultados del coeficiente kappa son las siguientes: menor de 0, no hay concordancia; 0,00 a 0,20, concordancia pobre; 0,21 a 0,40, concordancia débil; 0,41 a 0,60, concordancia moderada; 0,61 a 0,80, concordancia considerable (buena); y 0,81 a 1,00, concordancia casi perfecta (muy buena).

Para determinar la relación entre la capacidad de formación de biopelículas por ambos métodos y las variaciones de los medios de cultivo, se utilizó la prueba de ji al cuadrado (c^2^), con corrección de Yates en los casos necesarios; el nivel de significancia fue de 95 % y un valor de p≤0,05. Todos los análisis se realizaron con el programa Statgraphics Centurion XVII.

Se contó con el consentimiento informado de los pacientes para la realización del estudio.

## Resultados

### Distribución de los aislamientos bacterianos y fúngicos

Se procesaron 211 aislamientos clínicos de diferentes muestras biológicas en humanos (heces, orina, sangre, secreciones y tejidos). Los aislamientos bacterianos fueron 103, e incluyeron *K. pneumoniae* (n=28), *P. aeruginosa*, *S. aureus* y estafilococos coagulasa negativos (n=25 de cada uno). Los aislamientos fúngicos fueron 108, constituidos por el complejo *C. glabrata* (n=25), complejo *C. parapsilosis* (n=27), *C. albicans* y *C. tropicalis* (n=28 de cada uno).

*Microtitulación con cristal violeta en aislamientos bacterianos*. Los resultados con este método se obtuvieron utilizando caldo del medio infusión cerebro-corazón, que corresponde al método de referencia ^3^^,^^10^, y los caldos Müeller-Hinton y tripticasa de soya, propuestos en la presente investigación (cuadro 1). En general, los aislamientos bacterianos fueron poco formadores de biopelículas, con porcentajes de 71,8, 70 y 68 % en los medios infusión cerebro-corazón, tripticasa de soya y Müeller-Hinton, respectivamente. Se observó que *K. pneumoniae* tiene mayor capacidad de formar biopelículas respecto a los demás microrganismos bacterianos examinados, situación que puede estar influenciada por el lugar de procedencia del aislamiento o por la composición de su pared celular, rica en lipolisacáridos y que, a su vez, actúa como un mecanismo de resistencia contra elementos externos como los antimicrobianos ^3^.

**Figura 1:**
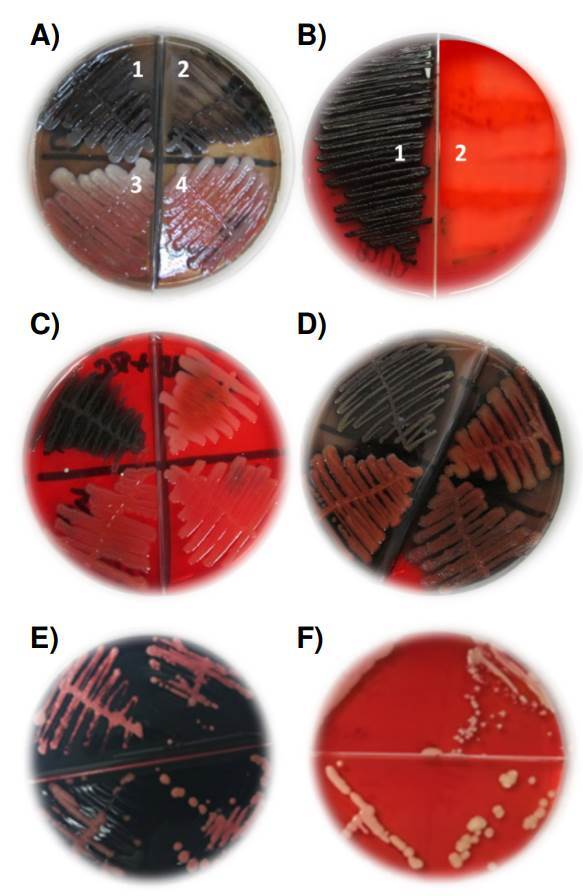
Presencia o ausencia de exopolisacáridos como indicadores de biopelículas, según el método de cultivo en agar con rojo Congo. A) *Pseudomonas aeruginosa*. Las cepas 1 y 2 fueron positivas y con precipitado de color negro, mientras que las cepas 3 y 4 fueron negativas y sin precipitado negro. B) *Klebsiella pneumoniae*. La cepa 1 fue positiva con precipitado negro y la cepa 2 fue negativa y sin precipitado negro. C) *Staphylococcus aureus*. La cepa 1 fue positiva y con precipitado negro, mientras que las cepas 2, 3 y 4 fueron negativas y sin precipitado negro. D) *Estafilococos coagulasa* negativa. La cepa 1 fue positiva y con precipitado negro, y las cepas 2, 3 y 4 fueron negativas y sin precipitado negro. E) Las cepas 1 y 2 de *Candida albicans*, la cepa 3 de *C. tropicalis* y la cepa 4 de *C. parapsilosis*, positivas y con color rosado intenso a fucsia. F) Las cepas 1 y 2 de *C. albicans*, la cepa 3 de *C. tropicalis* y la cepa 4 de *C. parapsilosis*, fueron negativas y con color blanco crema.

*Cultivo en agar con rojo congo en aislamientos bacterianos*. Mediante este método, los microrganismos bacterianos fueron capaces de exhibir su matriz de exopolisacáridos en un 63,5, 65 y 56 %, en los medios de infusión cerebro-corazón, tripticasa de soya y Müeller-Hinton, respectivamente. Se observó que los aislamientos de *P. aeruginosa* fueron poco formadores de biopelículas en los medios seleccionados, con respecto a los demás microorganismos evaluados (cuadro 1) (figura 1).

**Cuadro 1:** Formación de biopelículas en bacterias (grampositivas y gramnegativas) mediante los métodos de microtitulación con cristal violeta y agar con rojo Congo.

Microorganismo	Capacidad de formación de biopelículas en bacterias gramnegativas y grampositivas (n=103)																	
	Método por microtitulación con cristal violeta												Método en agar con rojo Congo					
	BHI			TBS				MH				BHI			TCB			MH
	NF	PF	MF	FF	NF	PF	MF	FF	NF	PF	MF	FF	N	P	N	P	N	P
*Klebsiella pneumoniae* (n=28)	3	25	0	0	5	23	0	0	8	20	0	0	5	23	3	25	9	19
*Pseudomonas aeruginosa* (n=25)	10	15	0	0	11	14	0	0	10	15	0	0	15	10	13	12	15	10
*Staphylococcus aureus* (n=25)	9	16	0	0	7	18	0	0	9	16	0	0	10	15	10	15	12	13
*Estafilococos coagulasa* negativa (n=25)	7	18	0	0	8	17	0	0	6	19	0	0	8	17	10	15	9	16

BHI: infusión cerebro-corazón; TBS: tripticasa de soya; MH: Müeller-Hinton; NF: no formadora de biopelículas; PF: poco formadora de biopelículas; MF: moderadamente formadora de biopelículas; FF: fuertemente formadora de biopelículas; N: negativo; P: positivo

*Microtitulación con cristal violeta en aislamientos del género Candida*. Las especies de *Candida* evaluadas de manera global fueron poco formadoras de biopelículas en un 86 y 87 %, en los medios de Sabouraud dextrosa correspondiente al método de referencia ^4^ y el caldo del medio infusión cerebro-corazón propuesto en esta investigación, respectivamente. No hubo variabilidad en el número de los aislamientos, aunque *C. albicans* pareciera ser menos formadora de biopelículas en ambos medios (cuadro 2).

**Cuadro 2:** Formación de biopelículas en Candida spp., mediante los métodos de microtitulación con tinción con cristal violeta y cultivo en agar con rojo Congo

Microorganismo	Capacidad de formación de biopelículas en Candida spp. (n=108)											
	Método por microtitulación con cristal violeta								Método en agar con rojo Congo			
	SDG				BHI				SDG		BHI	
	NF	PF	MF	FF	NF	PF	MF	FF	N	P	N	P
*Candida albicans* (n=28)	7	21	0	0	8	20	0	0	7	21	8	20
*Candida tropicalis* (n=28)	4	24	0	0	3	25	0	0	5	23	5	23
*Candida glabrata* (n=25)	1	24	0	0	2	23	0	0	1	24	2	23
*Candida parapsilosis* (n=27)	3	24	0	0	1	26	0	0	3	24	1	26

SDG: Sabouraud dextrosa; BHI: infusión de cerebro-corazón; NF: no formadora de biopelículas; PF: poco formadora de biopelículas; MF: moderadamente formadora de biopelículas; FF: fuertemente formadora de biopelículas; N: negativo; P: positivo.

*Cultivo en agar con rojo Congo en aislamientos del género Candida*. Los aislamientos de estas especies de Candida fueron capaces de exponer su matriz de exopolisacáridos en un 85 % de las colonias, en los dos medios utilizados (cuadro 2) (figura 1).

Las cepas de referencia ATCC®, evaluadas como control positivo en la presente investigación, fueron formadoras moderadas de biopelículas mediante la microtitulación con cristal violeta, y fueron positivas para la formación de matriz de exopolisacáridos mediante el cultivo en agar con rojo Congo.

*Comparación estadística mediante microtitulación con cristal violeta en aislamientos bacterianos*. Al evaluar la formación de biopelículas usando los caldos Müeller-Hinton y tripticasa de soya en lugar del medio infusión cerebro-corazón en el método de referencia semicuantitativo con cristal violeta, se obtuvo que el caldo Müeller-Hinton no tuvo un desempeño comparable al medio infusión cerebro-corazón (no existe asociación significativa, p≥0,05), mientras que el caldo tripticasa de soya sí tuvo un desempeño similar al medio infusión de cerebro-corazón (existe asociación significativa, p≤0,05). Sin embargo, el coeficiente kappa indicó que la concordancia categórica entre el desempeño de los caldos infusión de cerebro-corazón versus Müeller- Hinton (0,125) e infusión de cerebro-corazón versus tripticasa de soya (0,342), es pobre y débil, respectivamente (cuadro 3A).

**Cuadro 3:** Valoración de la concordancia y la relación de la capacidad de formación de biopelículas según los diferentes medios de cultivo utilizados: con cristal violeta o con rojo Congo.

(A) Método de Stepanovic con BHI Vs. Método de Stepanovic con sustitución de medios de cultivo MH y TSB en aislamientos bacterianos (n=103).Microtitulación en placa con cristal violeta							
M/C	S	E	VPP	VPN	K	*χ* ^2^	p
MH	71,6	41,4	75,7	36,4	0,125	1,617	0,2035
TSB	79,7	55,2	81,9	51,6	0,342	12,064	0,0005
(B) Método de Stepanovic con BHI Vs. Rojo Congo con sustitución de medios de cultivo (RC-MH y RC-TSB) en aislamientos bacterianos (n=103)							
M/C	S	E	VPP	VPN	K	*X* ^2^	p
RC-BHI	79,7	79,3	90,7	60,5	0,539	31,193	0,0001
RC-MH	59,6	51,7	75,8	33,1	0,096	1,059	0,6583
RC-TSB	77	65,5	85	52,8	0,396	16,587	0,0001
(C) Método de Stepanovic con BHI Vs. Método de Stepanovic con sustitución de medio de cultivo SDG. Género *Candida* (*C. albicans*, *C. tropicalis*, *C. glabrata* y *C. parapsilosi*s) (n=108)							
M/C	S	E	VPP	VPN	K	*χ* ^2^	p
SDG	97,8	80	96,8	85,4	0,801	62,655	0,0001
(D) Método de Stepanovic con BHI Vs. Rojo Congo con sustitución de medios de cultivo (RC-SDG y RC-BHI) Género *Candida* (*C. albicans*, *C. tropicalis*, *C. glabrata* y *C. parapsilosis*) (n=108)							
M/C	S	E	VPP	VPN	K	*χ* ^2^	p
RC-SDG RC-BHI	94,6	73,3	95,6	68,7	0,661	42,036	0,0001

BHI: infusión cerebro-corazón; TBS: tripticasa de soya; MH: Müeller-Hinton; SDG: Sabouraud dextrosa; RC: rojo Congo; S: sensibilidad; E: especificidad; VPP: valor predictivo positivo; VPN: valor predictivo negativo; K: coeficiente kappa; χ^2^: ji al cuadrado; p: valor de p

*Comparación estadística mediante cultivo en agar con rojo Congo en aislamientos bacterianos*. Se obtuvo que el agar Müeller-Hinton con rojo Congo tuvo menor desempeño frente al método de referencia con cristal violeta en caldo de medio infusión cerebro-corazón (no existe asociación significativa, p≥0,05), mientras que los agares tripticasa de soya e infusión cerebro-corazón tuvieron un desempeño similar al método de referencia al evaluar la formación de exopolisacáridos como indicadores de la formación de biopelículas (existe asociación significativa, p≤0,05). El coeficiente kappa indicó la concordancia categórica obtenida entre el desempeño del método semicuantitativo con cristal violeta versus el cualitativo con rojo Congo. La concordancia usando el agar de infusión de cerebro-corazón fue moderada (0,539), con el agar Müeller-Hinton fue pobre (0,096) y con el agar de tripticasa de soya fue débil (0,396) (cuadro 3B).

*Comparación estadística mediante microtitulación con cristal violeta en aislamientos del género Candida.* Al comparar el método de referencia semicuantitativo de microtitulación con cristal violeta utilizando el caldo infusión cerebro-corazón versus la sustitución por el caldo Sabouraud dextrosa, se obtuvo que ambos medios de cultivo tienen un desempeño similar (existe asociación significativa, p≤0,05). El coeficiente kappa indicó que la concordancia categórica entre ambos medios de cultivo es considerable o buena (cuadro 3C).

*Comparación estadística mediante cultivo en agar con rojo Congo en aislamientos del género Candida*. Se encontró que tanto el agar infusión cerebro-corazón como el Sabouraud dextrosa tuvieron un desempeño comparable al del método de referencia con cristal violeta y caldo de medio infusión de cerebro-corazón, en la producción de exopolisacáridos como indicadores de la formación de biopelículas en levaduras del género *Candida* (existe asociación significativa, p≤0,05). El coeficiente kappa indicó que la concordancia categórica entre el método semicuantitativo con infusión de cerebro-corazón *versus* el cualitativo con rojo Congo, usando los dos tipos de agar (infusión cerebro-corazón y Sabouraud dextrosa) es buena o considerable (0,661) (cuadro 3D).

## Discusión

En el presente estudio se pudo detectar la formación de biopelículas mediante dos métodos: uno considerado como de referencia (semicuantitativo de microtitulación con cristal violeta) y otro capaz de determinar la formación de biopelículas de forma indirecta, mediante la producción de exopolisacáridos (cualitativo de cultivo en agar con rojo Congo). En ambos se utilizaron cuatro géneros bacterianos y cuatro especies del género *Candida*, y se propusieron diferentes medios de cultivo para comparar con el método de referencia (microtitulación con cristal violeta en caldo infusión de cerebro-corazón), con la finalidad de proporcionar alternativas de medios de cultivo para ejecutar de rutina estos métodos en el laboratorio de microbiología. Con este diseño experimental, se obtuvo información novedosa, difícil de comparar con la información disponible en la literatura científica.

La evaluación de la producción de biopelículas en aislamientos bacterianos con el método semicuantitativo (cristal violeta), indicó que no es recomendable sustituir el caldo del medio infusión cerebro-corazón por los de tripticasa de soya y Müeller-Hinton, debido a que el desempeño de estos dos últimos medios fue pobre y débil, respectivamente.

Resultados similares se obtuvieron al comparar el método de referencia (cristal violeta en infusión de cerebro-corazón) con el método cualitativo con rojo Congo: sólo el agar infusión de cerebro-corazón presentó una concordancia moderada, lo que indica que este último puede utilizarse como método de tamizaje para la detección temprana de biopelículas en aislamientos bacterianos. Sin embargo, los resultados negativos obtenidos con este método deben confirmarse con el método de referencia.

Según lo reportado, el medio de cultivo más utilizado y que arroja mejores resultados al evaluar la producción de biopelículas mediante los métodos semicuantitativo (cristal violeta) y cualitativo (rojo Congo), es la infusión cerebro-corazón, seguido del tripticasa de soya ^3^^,^^4^^,^^9^^,^^12^^,^^16^. En esta investigación, se pudieron comprobar los resultados con el medio infusión cerebro-corazón, mas no con la tripticasa de soya. El uso del medio de cultivo Müeller-Hinton, no referido en la literatura consultada, fue seleccionado en este estudio como otra opción para el análisis de las biopelículas en el laboratorio de microbiología. Sin embargo, los resultados obtenidos desaconsejan su uso para estos fines.

En 2017, da Costa Lima *et al*. investigaron la capacidad de formar biopelículas de 20 cepas de *P. aeruginosa*. Mediante el método cualitativo de cultivo en agar con rojo Congo, detectaron que el 15 % de las cepas formaba biopelículas, mientras que, con el método de referencia semicuantitativo de microtitulación con cristal violeta identificaron lo mismo en el 75 % de las cepas ^15^. En dicho estudio, los investigadores suplementaron los medios de cultivo, para ambos métodos, con sacarosa, diferente al azúcar utilizado en la presente investigación que fue dextrosa. Según los resultados obtenidos en nuestro estudio, la dextrosa facilitó la formación de biopelículas con los aislamientos bacterianos debido, probablemente, al uso eficiente de este azúcar. Para mejorar la fórmula de preparación de los medios de cultivo, podrían evaluarse los azúcares que componen la matriz de polisacáridos de cada microorganismo ^20^.

En otras investigaciones se han obtenido resultados diferentes a los del presente estudio con el medio de cultivo tripticasa de soya. Hassan *et al*. evaluaron 110 aislamientos bacterianos grampositivos y gramnegativos, comparando los métodos expuestos anteriormente con cristal violeta y rojo Congo, y utilizando el medio de cultivo tripticasa de soya con glucosa y sin ella. Los investigadores obtuvieron 70 cepas productoras de biopelículas con el método semicuantitativo de microtitulación con cristal violeta y sólo cuatro cepas productoras con el método cualitativo de cultivo en agar con rojo Congo ^14^. Mathur *et al*. analizaron 152 aislamientos clínicos de *Staphylococcus* spp., comparando su capacidad de formar biopelículas por los dos métodos mencionados y usando como medio tripticasa de soya, con glucosa y sin ella. De los aislamientos evaluados con tripticasa de soya más glucosa, el 53,9 % resultaron productores, mientras que, de los aislamientos analizados con tripticasa de soya sin glucosa, solo el 4,6 % formó biopelículas a las 24 horas de incubación. Los investigadores concluyeron que el modificar la composición de los medios de cultivo ayuda a mejorar la precisión en la detección de las biopelículas ^21^.

Moreno *et al*. publicaron un estudio, en el cual evaluaron la capacidad de formación de biopelículas de 38 cepas de *K. pneumoniae* mediante los métodos semicuantitativo (cristal violeta) y cualitativo (rojo Congo), utilizando el medio de infusión cerebro-corazón. Los autores concluyeron que el método cualitativo de cultivo en agar con rojo Congo puede utilizarse en la tamización inicial para determinar la formación de biopelículas; no obstante, los resultados negativos deben confirmarse con el método de microtitulación con cristal violeta ^3^.

En la investigación de Oliveira *et al*., relacionado con la formación de biopelículas en bacterias grampositivas, se evaluaron 100 aislamientos de estafilococos coagulasa negativos en pacientes recién nacidos. Se compararon las metodologías semicuantitativa (cristal violeta) y cualitativa (rojo Congo), y se utilizó como método de referencia la reacción en cadena de la polimerasa (PCR) para detectar la presencia de los genes *icaA*, *icaC* e *icaD*, relacionados con la producción de biopelículas de los estafilococos. Hubo amplificación de estos genes en el 82 % de las cepas, mientras que, por los métodos semicuantitativo de microtitulación con cristal violeta y cualitativo de cultivo en agar con rojo Congo, el 81 y 73 % de las cepas formaron biopelículas, respectivamente. Ambos métodos alcanzaron una concordancia moderada con los resultados de la PCR, por lo cual los autores concluyeron que el método cualitativo con rojo Congo es confiable, sencillo y rápido para la detección de biopelículas, resultado comparable al del presente estudio ^13^.

En otro estudio comparativo, Achek *et al*. evaluaron la formación de biopelículas en 55 cepas de *S. aureus* con los dos métodos utilizados en la presente investigación. Se obtuvo que, con la microtitulación con cristal violeta, el 41,8 % de las cepas evaluadas producían biopelículas comparado con el 70,9 % obtenido con el método cualitativo con rojo Congo ^16^. Con este último método, se superó la detección de cepas formadoras de biopelículas con respecto al método de referencia con cristal violeta.

La investigación con los métodos descritos (semicuantitativo con cristal violeta y cualitativo con rojo Congo), mostró que el medio Sabouraud dextrosa puede sustituir al de infusión cerebro-corazón para evaluar la capacidad de las levaduras de formar biopelículas y funcionaría como medio de cultivo alternativo de rutina en el laboratorio de microbiología en caso de no contar con el de infusión cerebro-corazón. Sin embargo, los autores recomiendan que, si se obtienen resultados negativos, los mismos deben confirmarse utilizando el método de referencia de microtitulación con cristal violeta.

Moreno *et al*. desarrollaron una investigación con 30 cepas de *C. parapsilosis*, *sensu stricto*, aisladas en sangre, para comparar el método de microtitulación con cristal violeta y el de cultivo con rojo Congo, utilizando el medio Sabouraud dextrosa ^4^. Estos investigadores reportaron que el 63 % de los aislamientos mostró formación de biopelículas, con el primer método y, el 50 %, con el segundo. La concordancia entre ambas metodologías fue del 66,3 % y no fue posible obtener una asociación estadística significativa. Aunque el valor de p fue muy cercano al objetivo propuesto (p=0,052), la significancia pudo perderse probablemente por el número de cepas evaluado. En el presente estudio, se aumentó considerablemente el número de cepas de levaduras (n=108) obteniendo resultados favorables, lo cual demuestra que el método de cultivo en agar con rojo Congo, en medio Sabouraud dextrosa, puede ser útil para la evaluación de la formación de biopelículas en levaduras del género *Candida*.

En otro estudio de Moreno *et al*. ^22^, se evaluaron 28 cepas de *C. albicans* aisladas de secreciones vaginales utilizando los mismos dos métodos, pero con el medio Sabouraud dextrosa. Se obtuvo una concordancia de casi el 99 %. Es posible que las similitudes encontradas entre las cepas evaluadas estén relacionadas con su capacidad particular de formar biopelículas y con el origen y el tipo de la muestra estudiada.

Con base en los hallazgos obtenidos en el presente trabajo, el método cualitativo de cultivo en agar con rojo Congo resulta prometedor para determinar la capacidad de formar biopelículas de los aislamientos bacterianos y de levaduras. Se recomienda utilizar como medio de cultivo el caldo infusión cerebro-corazón en caso de evaluar aislamientos bacterianos y, el agar de este mismo o el Sabouraud dextrosa, como sustituto para evaluar levaduras. Su uso como método de tamizaje obliga a utilizar el método de referencia semicuantitativo de microtitulación con cristal violeta cuando se obtengan resultados negativos. Debido a que el método cualitativo de cultivo en agar con rojo Congo no requiere de instrumentos, ni de insumos especializados, es un candidato óptimo para ser implementado de rutina en los laboratorios de microbiología, en los que se suelan aislar microorganismos considerados persistentes y de difícil abordaje terapéutico.
